# Alpha-Lipoic Acid Reduces Methemoglobin and Oxidative Imbalance in the Blood and Liver Induced by Dapsone in Mice: Molecular Mechanism of Antioxidant Action

**DOI:** 10.1021/acsptsci.5c00204

**Published:** 2025-06-13

**Authors:** Savio Monteiro dos Santos, Joni Tetsuo Sakai, Bruno Alexandre Quadros Gomes, Lisa Maria Mendes de Almeida Souza, Roseane Guimarães Ferreira, Kaio Murilo Monteiro Espíndola, Ana Flávia Oliveira Pampolha, Kelly Davis, Pamela Suelen da S. Seabra, Larissa de N. da Paz Lopes, Fabricia de Jesus Paiva da Fonseca Sizo, Agnaldo da Silva Carneiro, Michael D. Coleman, Marta Chagas Monteiro

**Affiliations:** † Postgraduate Program in Pharmaceutical Sciences, Faculty of Pharmacy, Federal University of Pará/UFPA, Rua Augusto Corrêa, 01, Bairro Guamá, 66075-110 Belém, Pará, Brazil; ‡ Postgraduate Program in Neuroscience and Cell Biology, Federal University of Pará/UFPA, Rua Augusto Corrêa, 01, Bairro Guamá, 66075-110 Belém, Pará, Brazil; § Postgraduate Program in Pharmacology and Biochemistry, Faculty of Pharmacy, Federal University of Pará/UFPA, Rua Augusto Corrêa, 01, Bairro Guamá, 66075-110 Belém, Pará, Brazil; ∥ Laboratory Immunology, Microbiology, and In Vitro Assays (LABEIM), Faculty of Pharmacy, Federal University of Pará/UFPA, 66075-110 Belém, Pará, Brazil; ⊥ Postgraduate in Biology of Infectious and Parasitic Agents, Federal University of Pará/UFPA, Rua Augusto Corrêa, 01, Bairro Guamá, 66075-110 Belém, Pará, Brazil; # Postgraduate Program in Medicinal Chemistry and Molecular Modeling, Faculty of Pharmacy, Federal University of Pará/UFPA, 66075-110 Belém, Pará, Brazil; ¶ College of Health and Life Sciences, 1722 Aston University, Aston Triangle, Birmingham B4 7ET, U.K.

**Keywords:** alpha lipoic acid, dapsone, methemoglobin, oxidative stress, hepatotoxicity

## Abstract

Dapsone (DDS) is a sulfone clinically used in the treatment of dermatological disorders, such as dermatitis herpetiformis and psoriasis, besides *Toxoplasma gondii*, *Pneumocystis carinii*, and *Mycobacterium leprae* infections. However, the chronic use of DDS can lead to adverse effects involving all organ systems, such as dapsone hypersensitivity syndrome, methemoglobinemia, hemolytic anemia, and liver injury. These effects probably occur due to the presence of its toxic metabolite DDS-NOH, which can generate reactive oxygen species (ROS), and iron overload, causing oxidative stress. In this sense, antioxidant compounds with chelating properties such as Alpha-lipoic acid (ALA) may be an interesting adjuvant therapy strategy in treating or preventing oxidative stress and adverse reactions related to DDS. This study showed that DDS 40 mg/kg increased the methemoglobin and induced oxidative stress in the erythrocytes and liver of mice. However, post-treatment with ALA 12.5 mg/kg was able to restore redox status and hepatic biomarkers in DDS-intoxicated animals. Thus, inhibiting the formation of methemoglobin and lipid peroxidation in the blood, as well as reducing iron accumulation and production of hepatic enzymes stimulated by DDS metabolism. In addition, the molecular docking shows that ALA in its oxidized form can inhibit DDS-NOH. These findings highlight the potential of ALA and its thiol derivatives as antioxidants in counteracting the harmful effects of DDS metabolites. However, further investigations are necessary to understand the therapeutic potential and antioxidant mechanisms of ALA and its derivatives for the development of new strategies to prevent or alleviate oxidative damage associated with DDS treatment.

## Graphical abstract

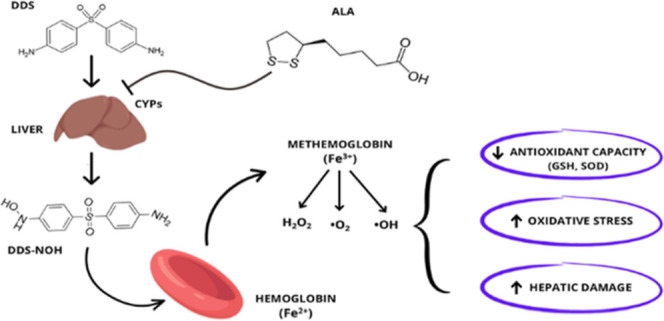

1

Dapsone (4,4′-diaminodiphenylsulfone, DDS) a sulfonamide compound widely used in the treatment of dermatological disorders, such as dermatitis herpetiformis and psoriasis, as well as infections caused by *Toxoplasma gondii*, *Pneumocystis jirovecii* pneumonia and it is commonly used for the treatment of leprosy.^1,2^ However, the chronic use of DDS can lead to adverse effects which involve several organs and dose-dependent adverse hematological reactions, such as dapsone hypersensitivity syndrome (DHS), methemoglobinemia, and possible liver injury.^3–6^ Orally ingested dapsone can follow two major metabolic pathways in the liver; first, through acetylation, forming monoacetyl and diacetyl dapsone, or second, when metabolized by N-hydroxylation through CYP2C9 and CYP2C19.^7^ The latter pathway produces toxic dapsone hydroxylamine (DDS-NOH), The main active metabolite implicated in the toxicity associated with DDS, including hemolytic anemia, agranulocytosis, methemoglobinemia, liver injury^8^ and formation of reactive oxygen species (ROS) in various cell types.^9^

Among the adverse effects of DDS, methemoglobinemia stands out clinically, as a hematological disorder where hemoglobin (Hb) cannot transport oxygen efficiently, leading to cyanosis. The formation of methemoglobin (MetHb) occurs because the ferrous iron (Fe^2+^) in Hb is oxidized to form ferric iron (Fe^3+^).^10,11^ Under normal conditions, Fe^3+^ is reduced to Fe^2+^ through cytochrome *b*5 and NADPH reductase pathways to maintain a methemoglobin level of <1%.^12^ Methemoglobinemia is related to oxidative stress, being an imbalance between oxidant ROS, reactive nitrogen species (RNS) and antioxidant (enzymatic and nonenzymatic) agents that result in the formation of oxidative biomolecules, including peroxidized lipids such as malondialdehyde (MDA).^13–15^ Furthermore, DDS can lead to an overload of free iron in the spleen and Kupffer cells in the livers of rats and humans, leading to hepatotoxicity.^10,13,16,17^

Currently, methylene blue (MB) remains the antidote of choice in the treatment of symptomatic methemoglobinemia, although it presents many adverse reactions.^18^ Alternatively, other agents could potentially alleviate methemoglobin’s impact by decreasing or blocking oxidative reactions. The antioxidant alpha lipoic acid (ALA), 1,2-dithiolano-3-pentanoic acid, has shown important antioxidant activity. ALA is insoluble in water and soluble in organic solvents, and it is found in most prokaryotic and eukaryotic microorganisms, as well as many plant and animal tissues.^19,20^ In the human body, ALA binds to lysine residues and acts as a cofactor in enzyme complexes involved in the metabolism of pyruvate oxidation pathways, the citric acid cycle, as well as the degradation and biosynthesis of amino acids, such as a-keto acid dehydrogenases and enzymes of the glycine cleavage system.^21–23^ Thus, current studies highlight ALA as a potential therapeutic agent in treating or preventing different pathologies related to oxidative stress.^24^ Thus, the aim of this study was to investigate the potential effects and molecular mechanisms of ALA on the formation of methemoglobin and oxidative stress in the plasma and liver of mice, induced by DDS treatment.

## 2 Materials and Methods

### 2.1 Ethics Statement

This study was carried out by the recommendations of the Guide for the Care and Use of Laboratory Animals of the Brazilian National Council for Animal Experimentation (http://www.sbcal.org.br/) and the NIH (Guidelines for the Care and Use of Laboratory Animals). The Institutional Animal Ethics Committee of the University of Pará/UFPA (CEUA), Protocol: 2411100816 and 2411100816 approved all procedures used in this study.

### 2.2 Mice

In the experiments, Adult male Swiss mice (7–8 weeks old, 20 g), used in the experiments, were obtained from the Evandro Chagas Institute (IEC-PA). Animals were kept in cages under controlled conditions of temperature (22 ± 3 °C), and light (12 h light/dark cycle) with food and water ad libitum, and acclimatized conditions for 3 days before use.

### 2.3 In Vivo Experimental Design

The mice were divided into 5 groups (*n* = 20 animals/group) according to the treatment schedule: (i) control group (animals treated with 0.9% saline solution and post-treated with DMSO 10%), (ii) DDS (animals treated with DDS (40 mg/kg), and post-treated with DMSO (10%)), (iii) and (iv) two groups with ALA (animals treated with DDS and post-treated with ALA at doses of 12.5 or 25 mg/kg, given 2 h after DDS), and to evaluate the percentage of methemoglobin in the blood, (v) animals were treated with DDS and post-treated with methylene blue-MB (1 mg/kg), an antidote used in the treatment of methemoglobin (positive control). All treatments were administered intraperitoneally (i.p.) for 5 consecutive days and each group was analyzed for blood parameters and other tissue.^25^ In the experiments, within 4 h after treatment, 10 animals from each group were euthanized by exsanguination and cardiac puncture to collect total blood from the aorta artery, along with tissues. The same procedure was repeated 24 h after treatment, where the other 10 animals from each group were euthanized to collect material for subsequent analysis. The mice organs were collected and stored at −80 °C for the tissue disruption process (obtaining the homogenate) and histological analysis, being used for laboratory analysis^26^ (Figure 1).

**Figure 1:**
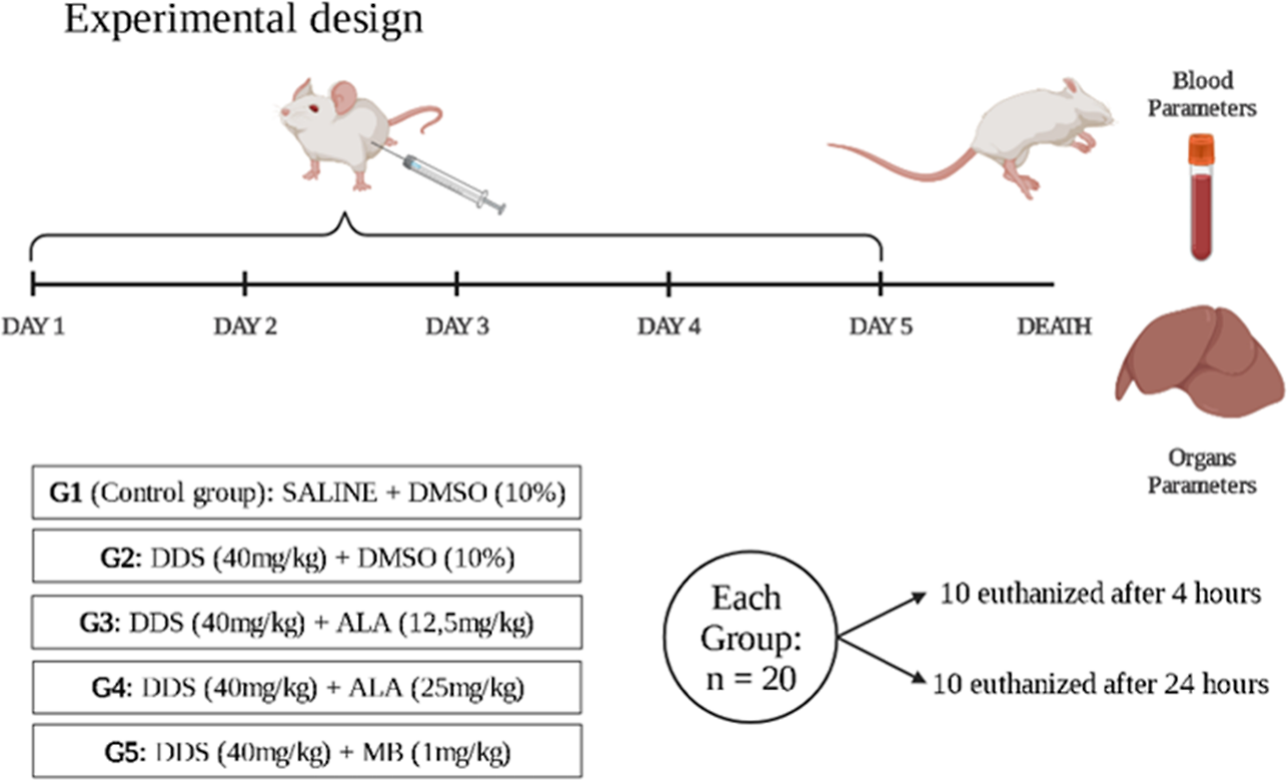
Experimental design of treatment in animals.

### 2.4 Determination of the Methemoglobin Percentage (MetHB)

The percentage of Methemoglobin in the blood of the animals was obtained by observing the absorbance variation caused by the addition of the neutralizing agent potassium cyanide (KCN) in a hemolysate buffered with potassium ferricyanide (K_3_Fe (CN)_6_) and another tube without this reagent. The tube containing hemolysate and K_3_Fe­(CN)_6_ was used as a standard, to convert all forms of Hb to MetHb. MetHb values below 2% were considered normal. Samples were read at 632 nm in a spectrophotometer.^27^

### 2.5 Determination of Trolox Equivalent Antioxidant Capacity (TEAC)

The total antioxidant capacity of blood and liver samples from mice was evaluated by Trolox-equivalent antioxidant capacity assay ((±) 6-hydroxy-2,5,7,8-tetramethyl chroman-2-carboxylic acid; Sigma-Aldrich; TEAC). In this assay, 2,2′-azino-bis (3-ethylbenzothiazoline-6-sulfonic acid) diammonium salt (ABTS) (Sigma-Aldrich) was incubated with potassium persulfate (Sigma-Aldrich) to produce ABTS^•+^, a green/blue chromophore. Inhibition of ABTS^•+^ formation by antioxidants in the samples were expressed as Trolox equivalents, determined at 740 nm.^28^

### 2.6 Determination of Superoxide Dismutase (SOD) Activity

The determination of SOD concentrations was evaluated in blood samples from mice, through the indirect detection of this enzyme that promotes 50% inhibition of the reduction of cytochrome C at 25 °C, pH 7.8, since SOD promotes the conversion of superoxide radical (^•^O_2_) into hydrogen peroxide (H_2_O_2_) and O_2_, preventing the reduction of cytochrome C, which is detected by spectrophotometry at 550 nm.^29^

### 2.7 Determination of Glutathione (GSH) Activity

The determination of GSH concentrations was performed in blood and liver samples from the animals, based on the ability of this antioxidant to reduce 5,5-dithiobis-2-nitrobenzoic acid (DTNB) (Sigma-Aldrich) to 5-thio-2-nitrobenzoic acid (TNB), and quantified by spectrophotometry at 412 nm.^30^

### 2.8 Determination of Lipid Peroxidation

Lipid peroxidation was measured in blood and liver samples from mice, as an indicator of oxidative stress, using the thiobarbituric acid reactive substance (TBARS) assay, where the reaction of malondialdehyde (MDA) and other substances with thiobarbituric acid (TBA; Sigma-Aldrich), at pH 2.5 at 94 °C, forms the pink colored MDA–TBA complex. The reading was performed in a spectrophotometer at 535 nm of absorbance.^31^

### 2.9 Analysis of Hepatic Biomarkers

The enzymes aspartate aminotransferase (AST) and alanine aminotransferase (ALT) were used as liver markers. These assays were carried out using commercial kits obtained from LABORCLIN (https://www.laborclin.com.br/)­(Ultraviolet UV kinetic methods and absorbance read at 340 nm). Also, the enzyme alkaline phosphatase (AP) was performed by a commercial kit obtained from Bioclin (https://www.bioclin.com.br/).

### 2.10 Determination of Serum Iron

Serum iron was measured in blood and liver samples using the modified Goodwin colorimetric method (Labtest kit), measured in a spectrophotometer at 560 nm. The results were expressed in μg/dL.

### 2.11 Histological Analysis

The liver sections of mice were fixed for 48 h in 10% formalin and were dehydrated by passing successfully successively in different mixtures of ethyl alcohol–water, washed in xylene, and embedded in paraffin. Sections of the liver (50 μm thick) were prepared and then stained with hematoxylin and eosin (H&E) for microscopic analysis in the 20× (A) and 40× objective (B). The liver sections of mice were observed for overfat deposition, microcystic steatosis, sinusoid congestion, hepatocyte ballooning, inflammation, and fibrosis.

### 2.12 Statistical Analysis

Statistical analysis was performed using GraphPad Prism 8 software (GraphPad Software Inc., La Jolla, USA). The data obtained were analyzed by analysis of variance (ANOVA) followed by Tukey’s test for multiple comparisons. Results were expressed as mean ± standard deviation and considered statistically significant for *p* ≤ 0.05.

### 2.13 Molecular Modeling

#### 2.13.1 Preparation of the Ligands and the Receptor

The three-dimensional structures of the molecules ligand DDS, DDS-NOH, and ALA were drawn with the Avogadro program,^32^ observing the configurations of the R-ALA and S-ALA isomers for the oxidized and reduced forms. The ligands were converted into more energetically stable structures through quantum energy minimization calculations using the Gaussian 03 program,^33^ with the DFT method and levels of the hybrid theory B3LYP^34^ and 6–31G (d, p) basis set.^35^

The three-dimensional structures of CYP-2C9, CYP-2C19, CYP-3A4, CYP-E21 receptors and hemoglobin used in computational experiments were retrieved from the Protein data bank,^36^ with the codes 4nz2, 4gqs, 7lxl, 3gph, and 2h35, respectively.^37^ Removal of water molecules and crystallization artifacts were performed using the Pymol.^38^ program. Polar Hydrogens and Gasteiger charges were added by AutoDock Tools (ADT) v1.5.6.^39^

#### 2.13.2 Molecular Docking

The molecular docking study was carried out with the Autodock 4.2.6/Vina program,^40^ with the objective of evaluating the intermolecular interactions between the ligands and the receptor molecules. The receptor molecules remained rigid and the ligands were allowed to rotate freely. All other parameters were kept as software defaults. The protein and ligand.pdb files were transformed into. pdbqt. A blind docking approach was applied to fit the ligands to the receptor, such that values of the central *XYZ* dimensions are described in Table 1.

**Table 1:** Central Dimensions and Size of the Grid Box for Carrying out Molecular Docking

	central dimensions			size		
systems	*X*	*Y*	*Z*	*X*	*Y*	*Z*
CYP-2C9	–60.971	–48.150	–23.431	30	30	30
CYP-2C19	–60.018	–46.715	–23.154	30	30	30
CYP-3A4	–19.776	–30.144	–10.464	30	30	30
CYP-E21	4.050	–3.907	–10.569	30	30	30
hemoglobin	–17.909	12.436	6.330	30	30	30

Each ligand molecule was coupled 104 times to the receptors using an exhaustivity value of 8. The ligand–receptor complex chosen for each system was the one with the lowest energy, in kcal mol^–1^, which shows the greatest chemical affinity. The final visualization of the anchored structure was performed using Discovery Studio Visualizer 2.5.^41^

## 3 Results

### 3.1 Evaluation of Biochemical and Oxidative Parameters in Blood

In this experimental study, blood samples showed high oxidative and biochemical parameters, which can occur in various organs and systems under different pathophysiological conditions, including the liver during DDS-induced toxicity. Thus, we evaluated the enzymatic and total antioxidant capacity, biomarkers of oxidative stress and liver histological changes caused by DDS, as well as the potential benefits of ALA to reverse oxidative stress and reduce liver histological changes induced by DDS.

To evaluate the oxidative effects of systemic DDS in an animal model, blood was collected 4 and 24 h after treatments, and the oxidative and antioxidant parameters were evaluated in the sample. Figure 2 shows that animals treated with DDS (40 mg/kg) and post-treated with DMSO significantly increased the percentage of methemoglobin (MetHb) formation at the times evaluated. In contrast, post-treatment with methylene blue (MB; 1 mg/kg) and ALA at doses of 12.5 and 25 mg/kg were able to inhibit DDS-induced MetHb formation. There was no difference in the percentage of MetHb when comparing the positive control (MB) and the ALA group at different doses. Furthermore, there was no significant difference in the inhibition of the MetHb percentage induced by ALA when comparing the two evaluated doses. Thus, the dose of 12.5 mg/kg of ALA was chosen to evaluate the other oxidative and antioxidant parameters in the blood and liver of the animals treated with DDS.

**Figure 2:**
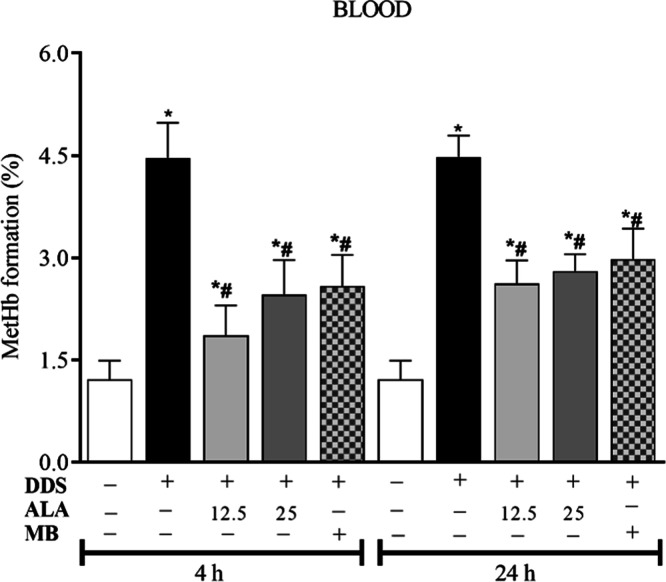
Effect of treatment with dapsone 40 mg/kg and post-treatment with ALA (12.5 and 25 mg/kg) or MB (1 mg/kg) on MetHb formation. *p ≤ 0.05 compared to the control group; #*p* ≤ 0.05 compared to the DDS group.

Figure 3 shows that DDS treatment induced a significant increase in blood iron (panel A) and MDA (panel B) levels at 4 and 24 h compared to the control group. However, post-treatment with ALA (12.5 mg/kg) was able to reverse the increase of iron and lipid peroxidation induced by DDS at the evaluated time intervals.

**Figure 3:**
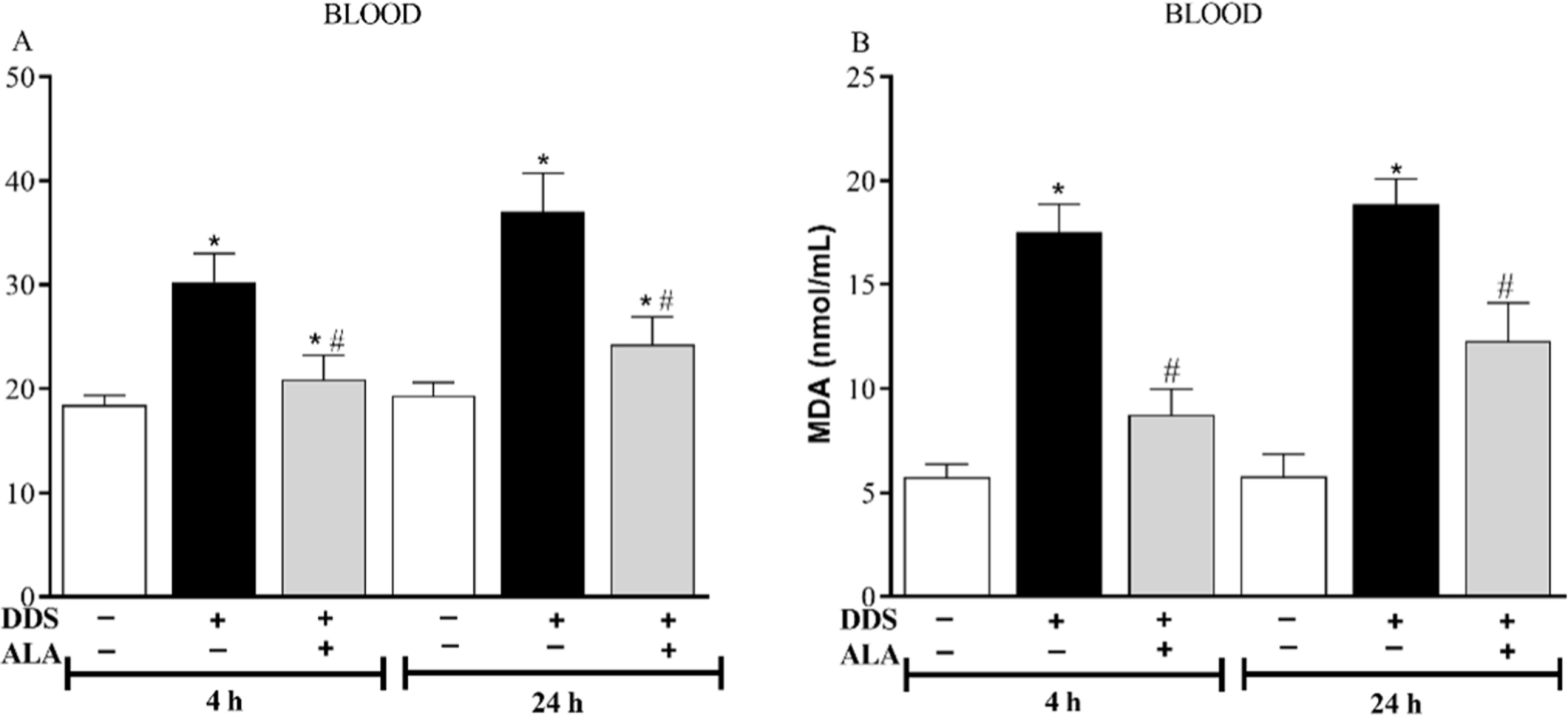
Effect of dapsone 40 mg/kg and post-treatment with ALA (12.5 mg/kg) on (A) iron (Fe) and (B) MDA levels in the blood. *p ≤ 0.05 compared to the control group; #*p* ≤ 0.05 compared to the DDS group.

Regarding the animals’ oxidant status, DDS treatment led to a decrease in most antioxidant parameters, including total antioxidant capacity (TEAC), glutathione (GSH), and superoxide dismutase (SOD) (Figure 4A–C), mainly 24 h after treatment. On the other hand, post-treatment with ALA was able to reverse the DDS-induced antioxidant inhibition, confirming the systemic protective effect of the antioxidant ALA in an animal model (Figure 4A–C).

**Figure 4:**
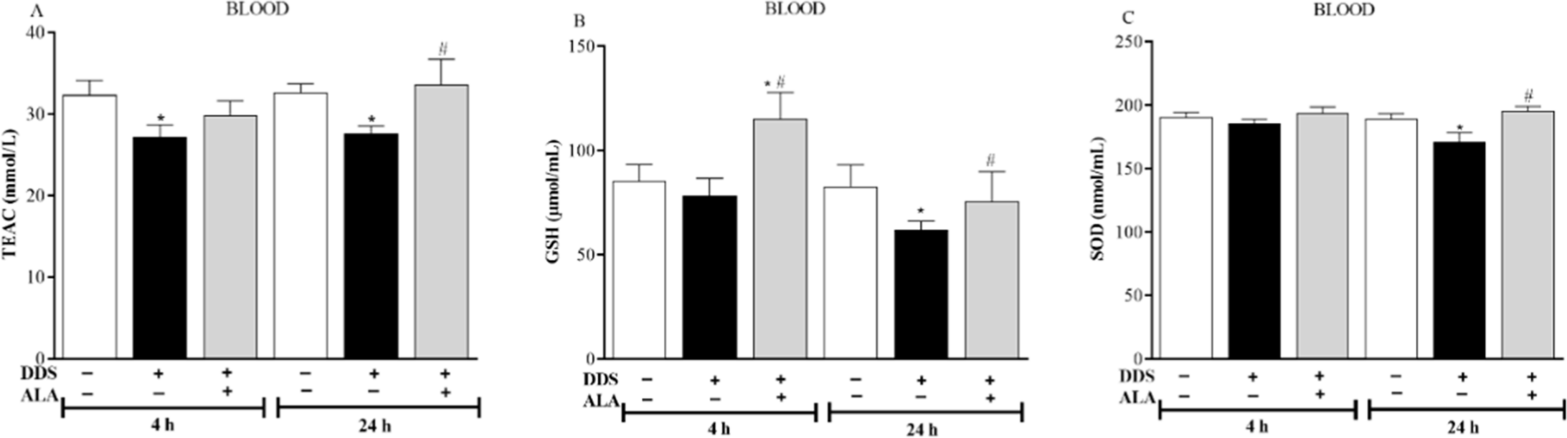
Effect of dapsone 40 mg/kg and post-treatment with ALA (12.5 mg/kg) on (A) total antioxidant capacity (TEAC), (B) GSH, and (C) SOD levels in the blood. *p ≤ 0.05 compared to the control group; #*p* ≤ 0.05 compared to the DDS group.

### 3.2 Evaluation of Biochemical and Oxidative Parameters in the Liver

Analysis of liver markers showed that DDS treatment significantly increased the levels of AST and Alkaline phosphatase (AP) in the liver, 4 or 24 h after treatment, respectively, compared to the control group (Figure 5A,C) but did not change ALT levels (Figure 5B). Post-treatment with ALA reversed the increase in AST and AP enzymes induced by DDS (Figure 5A,C), showing hepatic protection.

**Figure 5:**
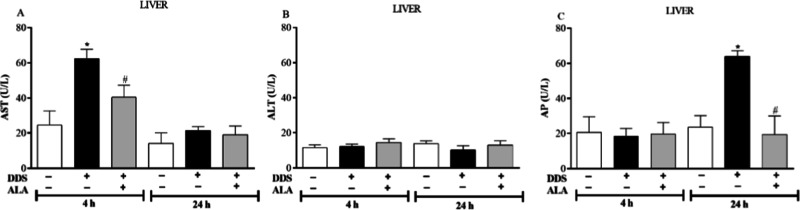
Effect of dapsone 40 mg/kg and post-treatment with ALA (12.5 mg/kg) for 5 consecutive days on (A) AST; (B) ALT; (C) AP in mice liver. *p ≤ 0.05 compared to the control group; #*p* ≤ 0.05 compared to DDS.

In the liver, DDS treatment led to oxidative stress with increased MDA and decreased total antioxidant capacity (TEAC) at 4 and 24 h after treatment (Figure 6A,B), and the drug also induced a decrease in GSH, and increased accumulation of iron at 4 h after treatment, compared to the control group (Figure 6C,D). In contrast, ALA reduced the levels of MDA and iron-induced DDS in the liver, mainly 4 h after treatment (Figure 6A,C). In addition, this antioxidant was able to reverse the decrease in TEAC induced by DDS, returning to control levels. ALA was also able to induce a significant increase in GSH, compared to the control and DDS-treated groups, at 4 and 24 h after treatment (Figure 6B,D). These data, taken together, show that this antioxidant exerts hepatoprotective and stimulating actions to the endogenous antioxidant capacity.

**Figure 6:**
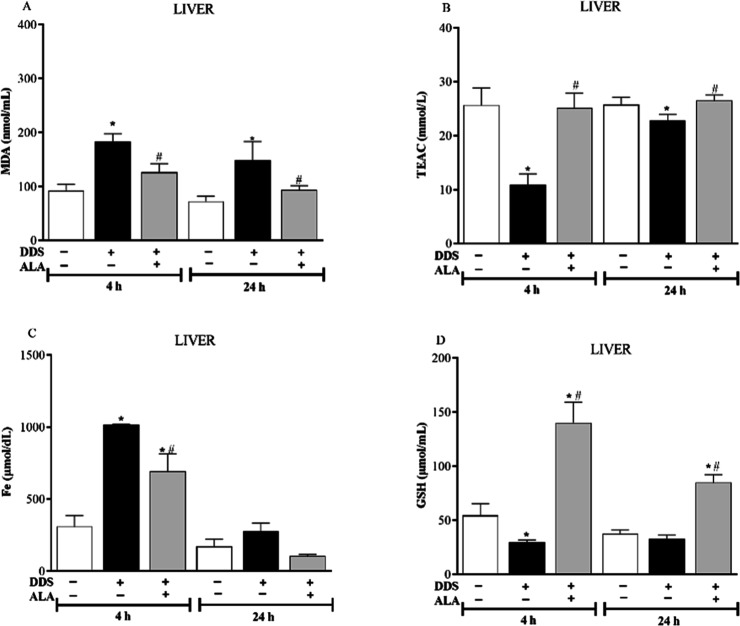
(A) MDA; (B) TEAC; (C) iron (Fe) (D) GSH concentrations in mice liver treated with DDS and post-treated with ALA (12.5 mg/kg) for 5 consecutive days. *p ≤ 0.05 compared to the control group and #*p* ≤ 0.05 compared to the DDS group.

To verify whether a higher dose of ALA (25 mg/kg) may have a toxic or protective action in the body, we treated the animals with ALA and/or DDS and evaluated the levels of lipid peroxidation in the liver, lung, and kidneys of these animals within 24 h after treatment. Figure 7 shows that treatment with ALA alone at a dose of 25 mg/kg did not alter MDA levels, and when associated with DDS it was able to reverse the increase in DDS-induced MDA formation in the liver, lung, and kidneys, thus confirming the protective effect of ALA in various organs.

**Figure 7:**
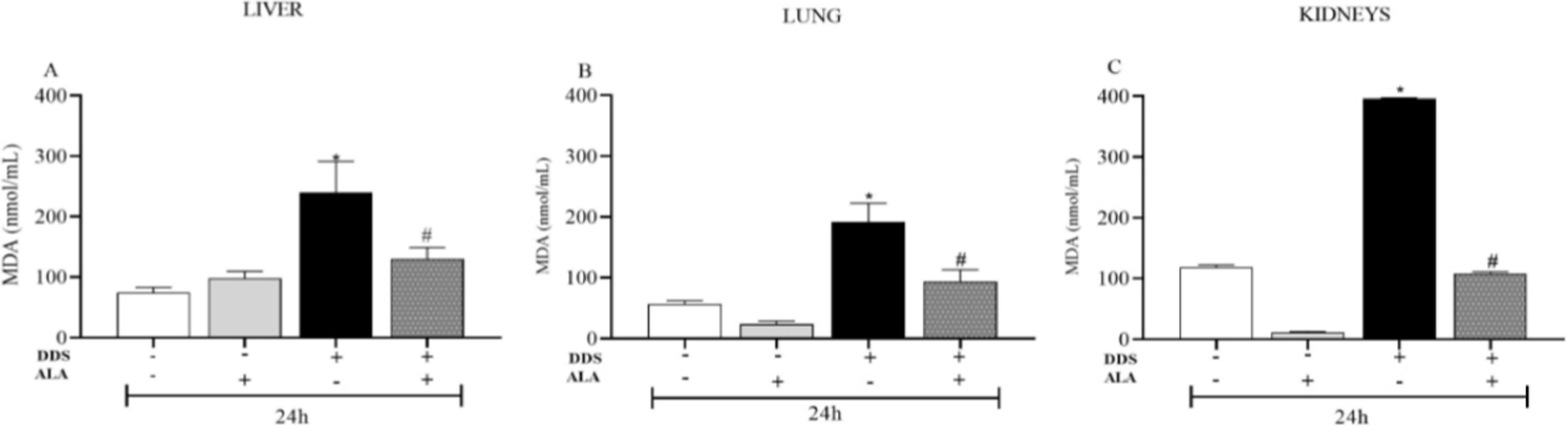
Effect of ALA (25 mg/kg) in animals treated or not with DDS (40 mg/kg) on MDA levels in liver (A) lung (B), and kidneys (C). *p ≤ 0.05 compared to the control group and #*p* ≤ 0.05 compared to the DDS group.

Histological analysis showed that DDS induces pathological changes in the liver. The liver sections of mice treated with saline (Figure 8A,D) showed a normal histological appearance. DDS treatment resulted in changes in liver architecture as indicated by focal necrosis, inflammatory cell infiltration, and steatosis (Figure 8B,E). Treatment with ALA (25 mg/kg) reduced histological alterations, inflammation, and steatosis compared to the DDS group (Figure 8C,F).

**Figure 8:**
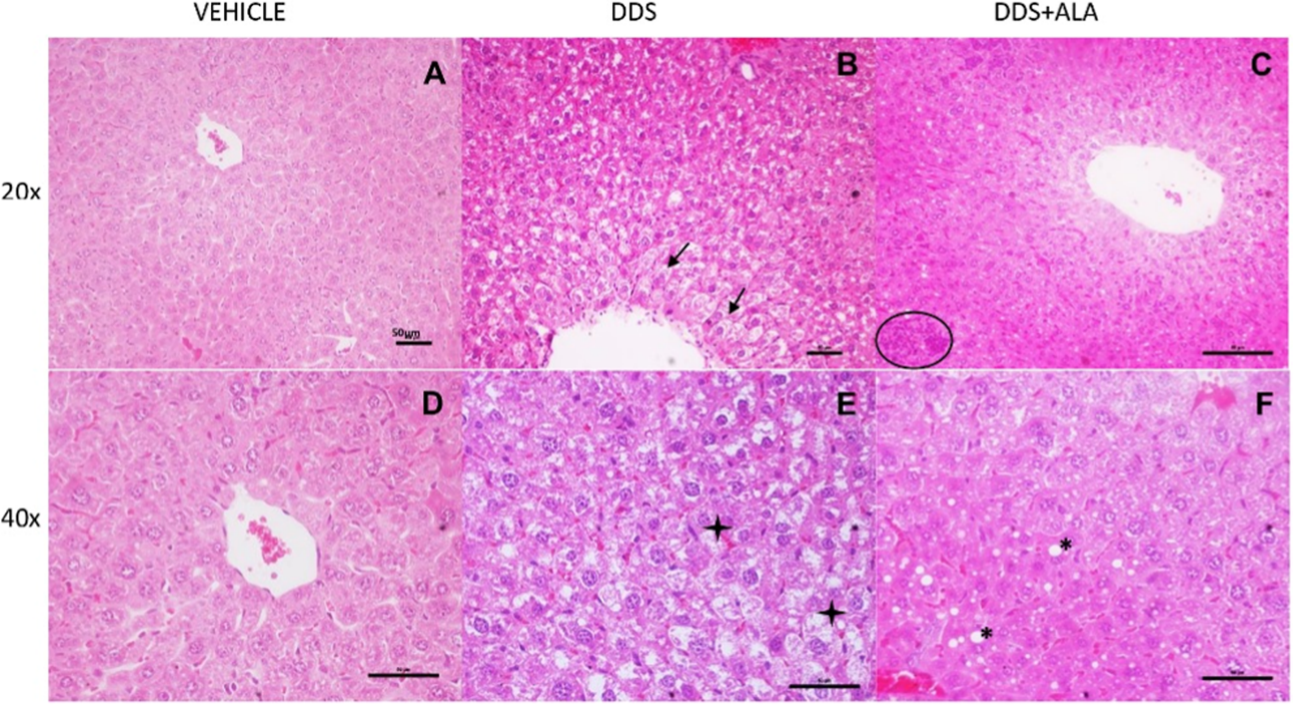
Liver sections of mice treated with saline in the 20× (A) and 40× (D); DDS (40 mg/kg) in the 20× (B) and 40× (E); DDS and ALA­(25 mg/kg) in the 20× (C) and 40× (F) stained with H&E. Overt fat deposition and microcystic steatosis (asterisk), sinusoid congestion (star), hepatocyte ballooning, inflammation (arrow and circles), and fibrosis, were detected in the naturally aged livers.

### 3.3 Theoretical Antioxidant Mechanism of ALA

The Molecular Docking Method is an effective and widely used tool to interpret the molecular aspects of ligand–protein interactions during new drug discovery. The molecular docking results reveal precious information in the context of the action of DDS on liver enzymes and its metabolite DSS-NOH, as well as ALA that acts as an inhibitor, considering its stereochemistry and its oxidized and reduced form. Furthermore, the behavior of these last two in coupling with the hemoglobin molecule was verified (Figure 9).

**Figure 9:**
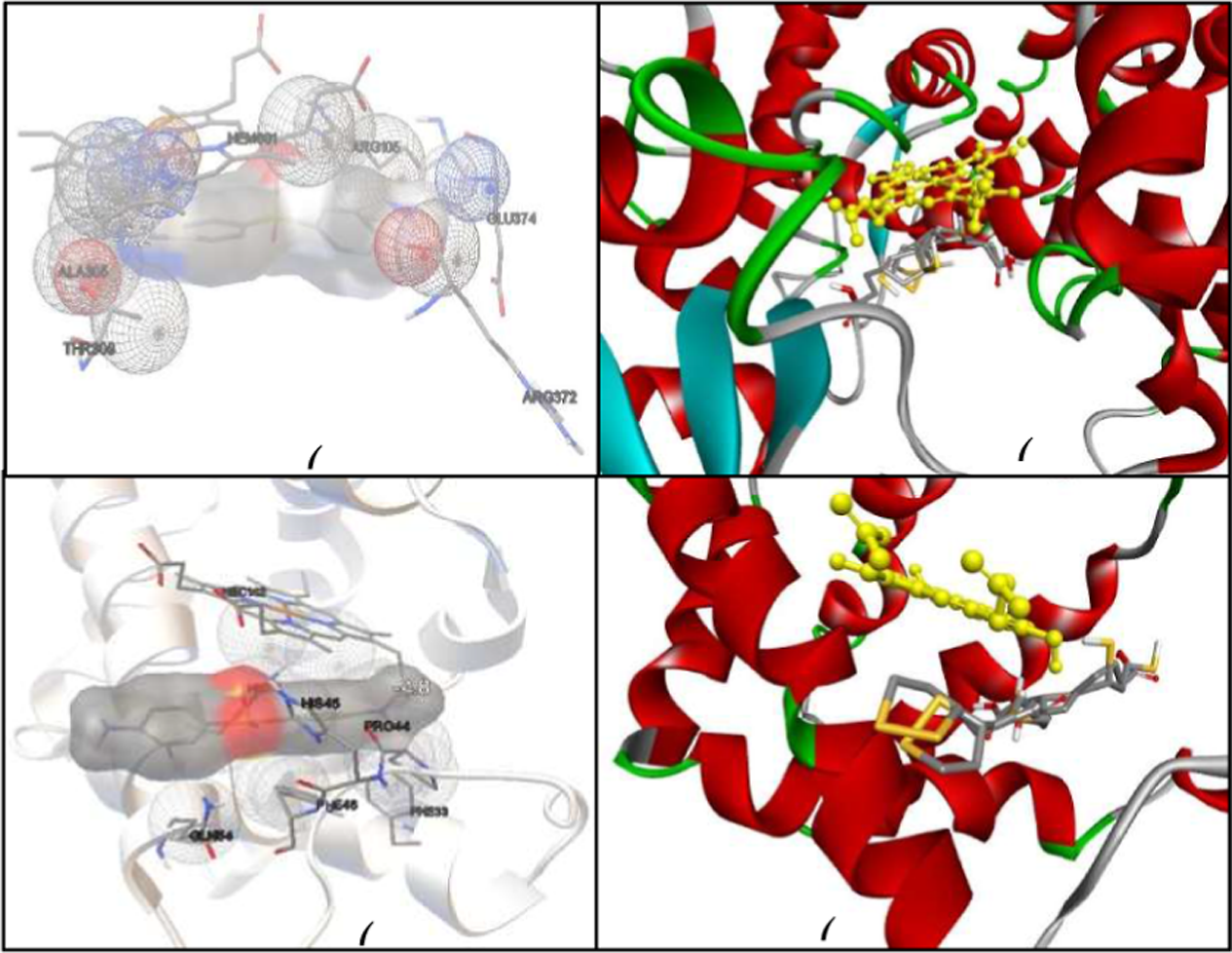
(a) Structure of CYP3A4 anchored with the DDS molecule. (b) Structure of CYP3A4 complexed with hydroxydapsone and the ALA ligand in the R–S configuration in the oxidized form (R-ALA and S-ALA) and in the reduced form (rALA, sALA). (c) Structure of hemoglobin anchored with the metabolite hydroxydapsone (d) Structure of hemoglobin complexed with the ALA ligand in the R-S configuration in the oxidized form (R-ALA and S-ALA) and in the reduced form (rALA, sALA).

The binding energy results, represented in Table 2, demonstrate that the DDS molecule acts more significantly on the hepatic enzymes CYP2C9, CYP2C19, CYP3A4 and CYPE21. Thus, ALA and its isomers are unable to act as inhibitors of DDS binding to the tested CYPs.

**Table 2:** Binding Energy, in kcal mol^–1^, Established between Biological Receptors: CYPs and Hemoglobin with DDS, DDS-NOH, R-ALA, rALA, S-ALA, and sALA Molecules

	DDS	R-ALA	rALA	S-ALA	sALA	DDS-NOH
CYP-2C9	–6.6	–5.0	–4.3	–5.0	–4.5	–4.3
CYP-2C19	–6.3	–4.5	–4.1	–4.6	4.2	–3.9
CYP-3A4	–7.2	–5.1	–4.5	–5.3	–4.7	–2.9
CYP-E21	–8.4	–6.5	–5.7	–6.5	–5.7	–4.3
hemoglobin		–5.4	–4.7	–5.3	–4.9	–4.8

Considering a competitive process, lower binding energy values indicate greater chemical affinity with the receptor systems, evidencing the preference of biological targets for DDS. In this context, an important finding must be considered. It is observed that the energy values of CYP2C9 and CYP2C19 are −6.6 and −6.3 kcal mol^–1^, while those of CYP3A4 and CYPE21 are −7.2 and −8.4 kcal mol^–1^, respectively. This difference in energy can be justified by the interaction that the DDS molecule carries out with the heme group of these last two systems, which is not observed for the first two. Furthermore, there is a slight difference in energy between CYP3A4 and CYPE21, this phenomenon can be explained by the type of interaction that DDS carries out with both systems. In CYP3A4, there are three hydrogen bonds and five pi-type bonds, while in CYPE21 there are eight pi-type interactions, showing that van der Waals interactions drive the metabolic process.

Regarding DDS-NOH, it is observed that, once present in liver enzymes, it is preferentially inhibited by the ALA molecule in its oxidized form. In all CYPs, DDS-NOH showed higher binding energy, indicating lower chemical affinity with these receptors. However, no significant difference in energies is observed when considering the R–S configurations, demonstrating that this chemical aspect is not relevant in the DDS-OH inhibitory process.

The most significant energy values are observed in CYP3A4 and CYPE21 with R-ALA = −5.1 and −6.5 kcal mol^–1^ and S-ALA = −5.3 and −6.5 kcal mol^–1^, respectively. The greater chemical affinities of CYPE21 can be explained by the nature of the interactions established between the ALA molecule and the biological system. Here it is again observed that inhibition is driven by pi-type interactions, characterizing a nonpolar chemical environment in the inhibitory process, characterizing a nonpolar chemical environment in the inhibitory process.

In hemoglobin, the DDS-NOH molecule is probably inhibited by the presence of the ALA system in the oxidized form with R or S configuration. The lowest energy values of R-ALA = −5.4 and S-ALA = −5.3 kcal mol^–1^ direct for possible inhibition against the DDS-NOH molecule, which has a binding energy value equal to −4.8 kcal mol^–1^.

## 4 Discussion

In clinical practice, DDS is associated with toxicity and significant adverse reactions. These adverse reactions include dapsone syndrome, characterized by symptoms such as fever, exfoliative dermatitis, lymphadenopathy, and lymphocytosis, as well as methemoglobinemia, hemolytic anemia, and hepatotoxicity, probably due to its metabolite DDS-NOH.^10,25^ This study demonstrated that the intraperitoneal administration of DDS 40 mg/kg disrupts the redox balance in the blood and liver, leading to hepatitis and steatosis in mice. This disruption is evident in an increase in methemoglobin, iron levels, and lipid peroxidation in the blood. Additionally, DDS administration resulted in a decrease in total antioxidants, including GSH and SOD. In the livers of mice, DDS treatment led to elevated levels of AST, AP, iron, and lipid peroxidation, while significantly reducing total antioxidants and GSH levels, inducing hepatotoxicity with hepatitis and steatosis. Furthermore, DDS administration caused an increase in MDA levels in other organs. In contrast, post-treatment with the antioxidant ALA was able to reverse the oxidative damage observed in the blood and liver. ALA effectively reduced methemoglobin and lipid peroxidation levels while increasing the levels of total antioxidants, including GSH and antioxidant enzymes. Furthermore, ALA administration mitigated iron accumulation and suppressed the elevation of liver enzymes and hepatic damage induced by DDS treatment in animals.

The half-life of dapsone in rodents, including male mice, ranges from 7.7 to 20 h.^44^ In humans, the half-life of dapsone is typically reported to be around 20 to 36 h.^10^ While the in vivo toxicity of dapsone has been studied in rats,^45^ it has been observed that in various species, including mice, the liver has the capacity to metabolize dapsone into dapsone-hydroxylamine (DDS-NOH), which can lead to toxicity.^46–48^ In our experimental model, the administration of DDS 40 mg/kg intraperitoneally was based on previous studies described by Ahmadi et al.^49^ and Bergamaschi et al.,^26^ which showed that the dose of 40 mg/kg (intraperitoneal) of DDS is known to induce methemoglobinemia in mice and rats. Methemoglobinemia is the most notable adverse effect observed in mice, with peak levels reaching within 0.5 to 1 h after dosing. However, complete recovery (methemoglobin reversal) occurs in 4 h.^49^

Our data showed that the administration of DDS 40 mg/kg induced an increase in the formation of MetHb at 4 and 24 h after 5 days of treatment. The ability of DDS to oxidize Hb is attributed to intracellular redox cycling reactions facilitated by its hydroxylated metabolite, DDS-NOH. This metabolite converts oxyhemoglobin and DDS-NOH to methemoglobin and nitroso DDS (DDS-NO), which is then reduced back to DDS-NOH by GSH. The reformed DDS-NOH can further oxidize oxyhemoglobin, leading to a futile cycle of oxidation.^45,50^ Studies by Coleman^47^ and Albuquerque^50^ have shown that the DDS-NOH metabolite at high concentrations (above 20–45%) causes methemoglobin formation in healthy erythrocytes during 1 h of incubation. Clinical studies conducted by Dalpino et al.^51^ and Schalcher et al.^13^ demonstrated that leprosy patients treated with DDS had higher levels of methemoglobin compared to healthy individuals, likely due to the oxidizing action and hemolytic effects of DDS. As a result, therapeutic alternatives, such as antioxidants like ALA and resveratrol, have been investigated to mitigate the toxic effects of DDS.

The biological activities of ALA are linked to its structure, as ALA has two sulfur atoms, one at carbon 6 and the other at carbon 8, forming two thiol groups (-SH), connected by a disulfide bridge. This disulfide bridge can be oxidized or reduced and forms covalent bonds with proteins. The oxidized form (ALA) can be rapidly reduced in the body, resulting in the formation of dihydrolipoic acid (DHLA). Additionally, ALA contains a single central chiral carbon at C6, which is asymmetric, leading to two optical enantiomers or stereoisomers: R-(+)-lipoic acid (R-ALA) and S-(−)-lipoic acid (S-ALA).^42,43^ In our theoretical study, the binding energy results demonstrate that the DDS has a great chemical affinity by CYP2C9, CYP2C19, CYP3A4 and CYPE21, more than ALA and its isomers. In addition, DDS-NOH is preferentially inhibited by the ALA in its oxidized form. DDS-NOH showed higher binding energy in all CYPs, thus lower chemical affinity.

The selection of ALA doses (12.5 and 25 mg/kg) in our study was based on previous studies conducted by Ledesma and Aragon^52^ and Takaoka et al.^53^ Our data suggest that both doses of ALA were effective in reversing the increase in DDS-induced methemoglobin (MetHb) formation at both 4 and 24 h after treatment. The inhibitory effect of ALA on MetHb formation was comparable to that of the antidote methylene blue, suggesting that ALA possesses a strong antioxidant capacity and can act on MetHb by reducing it to Hb or creating a microenvironment that prevents its oxidation. These findings are consistent with the results reported by Coleman and Walker,^54^ who observed a significant reduction in DDS-NOH-induced MetHb when ALA was incubated with erythrocytes from both diabetic and nondiabetic individuals in vitro.

Recently, we published a study demonstrating the protective action of ALA on the hemoglobin molecule and the reduction of oxidative stress induced by DDS-NOH in vitro, similar to the data obtained with animals treated with DDS. In this in vitro study, we demonstrated the efficacy of pretreatment with a racemic mixture of ALA and its enantiomers (R/S-ALA) in reducing the formation of methemoglobin induced by DDS-NOH in vitro. Additionally, ALA pretreatment led to an increase in GSH levels and provided protection to lymphocytes against DNA damage. These results highlight the remarkable effectiveness of this compound in protecting cells from oxidative mechanisms.^55^ Furthermore, through molecular docking studies, we elucidated differences in binding energies and interactions between the R-ALA and S-ALA isomers with Hb. Specifically, the R-ALA isomer exhibited a more stable conformation and interacted with the HEME group, whereas the S-ALA isomer interacted with the pro-44 amino acid residue. This disparity in binding properties elucidates the superior inhibitory effect of the R-ALA isomer on methemoglobin formation in vitro. These findings underscore the potential therapeutic utility of ALA, particularly the R-ALA isomer, in future treatments for individuals undergoing chronic dapsone therapy, including those afflicted with leprosy.^55^

In this regard, ALA possesses beneficial properties that contribute to its potential therapeutic effects. As an antioxidant, ALA can modulate cellular metabolism and aid in the elimination of toxic substances. It exhibits rapid uptake and is primarily metabolized in the liver, where it has been shown to promote tissue recovery from injury. ALA has also demonstrated efficacy in chelating transition metals and inhibiting the activation of NFκ-B, thereby blocking inflammatory responses.^56,57^ The antioxidant capacity of ALA is attributed to its ability to be endogenously reduced to a dithiol form called dihydrolipoic acid (DHLA), which is highly efficient in detoxifying oxidant radicals.^54,58^ These properties of ALA make it a valuable therapeutic agent with potential applications in various pathological conditions associated with oxidative stress and inflammation.

In this study, DDS treatment led to an increase in iron concentrations in the liver and blood. Iron plays a crucial role in the oxidation–reduction balance, and its concentration needs to be tightly regulated to prevent the generation of ROS such as the hydroxyl radical (^•^OH), which can cause cellular and tissue damage. The formation of methemoglobin (MetHb) and the release of heme and free iron into the blood can catalyze oxidative reactions that are potentially harmful. Our findings align with the experiments conducted by Ciccoli et al.,^17^ who demonstrated that acute intoxication of rats with DDS resulted in a significant increase in MetHb and Fe^3+^ levels. Under normal conditions, iron is bound to transferrin (Tf) in the plasma. Tf has the capacity to transport up to 12 mg of iron. However, in pathological situations, the transport capacity of Tf can be exceeded, leading to the accumulation of iron in its nontransferrin-bound iron (NTBI) form. NTBI acts as a catalyst for oxidative reactions, promoting the synthesis of superoxide (^•^O^2^) and hydroxyl radicals (^•^OH), which can potentially cause tissue damage.^59^ These findings highlight the importance of regulating iron levels and preventing iron overload to avoid oxidative damage and maintain the redox balance in the body.

Our results also suggest that ALA can decrease iron overload following DDS treatment at both the 4 h and 24 h time intervals. This indicates that ALA may possess iron-chelating properties, which can prevent Fenton and Haber–Weiss reactions and subsequently reduce the generation of ROS such as the ^•^OH. Previous in vitro studies on epithelial cell cultures have demonstrated that ALA treatment can lead to a decrease in iron absorption from transferrin and an increase in iron deposition in ferritin, indicating its potential role in modulating iron metabolism.^60^ Additionally, in a rat model of iron overload induced by ferrous sulfate treatment, ALA post-treatment resulted in a significant reduction (47%) in serum iron levels and a concomitant decrease (74%) in blood levels of MDA, a lipid peroxidation biomarker.^61^ Furthermore, ALA was found to mitigate the detrimental effects of iron-induced ROS, as evidenced by its ability to increase the integrity of the erythrocyte membrane, and decrease osmotic fragility, ultimately leading to a decrease in hemolysis. Taken together, these data support the notion that ALA exhibits beneficial antioxidant properties by reducing damage caused by ROS and iron overload. Its iron-chelating capacity may contribute to maintaining redox balance and protecting against oxidative stress-related damage.

The excessive production of ROS can lead to an increase in lipid peroxidation, resulting in the degradation of membrane phospholipids and the generation of toxic polyunsaturated fatty acids, which can cause cellular and organ damage.^62^ MDA is one of the main products of lipid peroxidation and serves as a widely used marker for assessing oxidative stress.^62–64^ In our study, the administration of DDS resulted in an elevation of MDA levels in the blood and various organs such as the liver, lung, and kidneys, indicating increased lipid peroxidation and oxidative stress. However, post-treatment with ALA was able to reduce lipid peroxidation and restore the redox balance in the tissues and cells. This suggests that ALA possesses potent antioxidant properties and can effectively mitigate oxidative damage caused by DDS treatment. Furthermore, in a study using male rats treated with intraperitoneal DDS, administration of 30 mg/kg resulted in an increase in the GSSG/GSH biliary output ratio, a sensitive indicator of oxidative stress, and in lipid peroxidation.^65^ Additional research conducted by El-Shenawy et al.^66^ demonstrated that post-treatment with ALA (16.12 mg/kg) was able to reverse hepatic oxidative stress induced by dimethyl nitrosamine in mice, as evidenced by the inhibition of MDA levels. Overall, the results from our study and other investigations indicate that ALA can effectively counteract oxidative stress, reduce lipid peroxidation, and contribute to the restoration of redox balance in tissues and cells affected by oxidative drugs or pathological conditions.

DDS treatment has been associated with liver damage, even at recommended doses, and it can persist in the body for an extended period, up to 35 days after administration.^67,68^ In our study, treatment with DDS resulted in increased levels of AST and AP in the liver of mice, indicating probable liver damage. However, post-treatment with ALA was able to reverse this damage. AST is a liver marker that has a relatively long half-life of approximately 17 h, which may explain the high levels observed at the 4 h time point after DDS treatment. However, post-treatment with ALA significantly reduced AST levels, suggesting a decrease in liver damage. This could be attributed to the regenerative properties of ALA, promoting the replenishment of GSH and other antioxidants that prevent excessive ROS production and inhibit oxidative damage. In addition, AP levels were found to be increased at the 4 h time point after DDS treatment. AP is used as a marker for cholestasis, which refers to the interruption of bile flow in the bile ducts. The inhibition of AP observed in the post-treatment with ALA may be attributed to the reduction in liver damage, which is facilitated by the regeneration of GSH and other antioxidants, as seen with AST. Overall, the administration of DDS resulted in liver damage, being evidenced by elevated AST and AP levels. However, post-treatment with ALA was able to reverse liver damage, likely through the regeneration of antioxidants and the prevention of oxidative damage.

The combined action of various enzymatic and nonenzymatic antioxidants presents in the body, including SOD, CAT, ceruloplasmin, transferrin, protein nonthiols, vitamins C and E, and uric acid, prevents the formation of free radicals and protects against cellular and tissue damage.^13^ In our study, it was observed that DDS treatment decreased TEAC in the blood and liver of mice, probably through consumption and saturation of the antioxidant system that is responsible for scavenging and neutralizing ROS. This suggests that metabolites of DDS have pro-oxidant properties and can disrupt the antioxidant balance in the body, leading to oxidative imbalance and potential damage. However, the administration of ALA was found to be effective in restoring antioxidant factors, including TEAC, as well as the activities of enzymatic antioxidants such as SOD, particularly within 24 h after DDS treatment. Thus, leading to the direct removal of ROS and other free radicals, providing an additional mechanism for mitigating oxidative stress. The effectiveness of ALA in restoring antioxidant parameters may be attributed to its pharmacokinetic characteristics and its ability to counteract the oxidative effects of DDS metabolites. In addition, this could be attributed to various factors such as the short duration of the study from treatment administration to the measurement of effects, the selection of an inappropriate target to measure oxidative stress, or the relatively small dose of antioxidants administered. It highlights the importance of considering factors such as treatment duration, appropriate markers of oxidative stress, and optimal dosing of antioxidants when studying their potential benefits in mitigating the oxidative effects of dapsone. These insights contribute to the overall understanding of the complexities involved in antioxidant therapy and emphasize the need for further research to optimize treatment approaches and maximize the benefits of antioxidants in the context of dapsone-induced oxidative stress.

Our data also analyzed GSH, demonstrating that DDS treatment decreased the concentrations of this tripeptide in the blood and liver of mice. In this sense, the decrease in GSH levels may be related to the consumption of GSH in the blood and the process of metabolizing DDS-NOH to DDS-nitrosoarene in the liver, which releases an electron to O^2^, thus forming the O^2•^, altering the cycle redox of glutathione and generating oxidative stress.^62,63^ The liver is the site responsible for the metabolism of numerous reactive metabolites such as DDS-NOH, which cause liver damage through the production of ROS.^69^ Radicals such as O^2•^, H^2^O^2^, and HO^•^ cause liver damage, necessitating their attenuation by the antioxidant system, whether enzymatic or nonenzymatic.^70,71^ The activity of GSH is complementary to glutathione peroxidase, which catalyzes H^2^O^2^ into water and O^2^, to limit the formation of the highly reactive HO^•^ radical.^72^

In this study, the treatment with DDS resulted in liver histological changes, such as acute hepatitis, inflammatory infiltrates, sinusoid congestion and steatosis. It may be due to the formation of ROS and oxidative stress induced by high levels of iron and ferroptosis. This oxidative process can accumulate hydroperoxides, which is associated with lipid peroxidation of membrane phospholipids, leading to injury and necrosis in hepatocytes.^73^ In addition, ALA (25 mg/kg) reduced the histological and inflammatory alterations induced by DDS treatment. This can be attributed to the antioxidant and chelating ability of ALA, which significantly reduces oxidative damage and leads to a reduction of pathological changes and restoring normal liver functions. Dulundu et al. demonstrate that lipoic acid treatment also reduces neutrophil accumulation, oxidative injury, and hepatic dysfunction, through its ability to inhibit neutrophil infiltration, balance oxidant-antioxidant status, and regulate the generation of inflammatory mediators.^74^ The cholestatic injury caused by dapsone and its N-hydroxylated metabolites hinders bile flow, leading to oxidative stress and hepatic necrosis. This process further results in hemolysis, which is responsible for hepatitis due to iron overload in the liver. Drug-induced liver injury is a significant cause of hepatotoxicity and presents challenging clinical problems in both diagnosis and management.^75^

According Ezhilarasan,^76^ the administration of DDS in humans and experimental animals has been shown to cause deviations from normal liver structure and function, resulting in oxidative stress, hepatic necrosis, cholestasis, hepatitis, and other issues. The hepatotoxicity of DDS appears to occur through several mechanistic pathways, including mild injury to hepatocytes and elevation of hepatotoxic marker enzymes, induction of oxidative stress by the DDS-NOH metabolite via the N-hydroxylation pathway, induction of cholestasis through bile duct and bile flow obstruction, cholangitis due to focal bile duct destruction, and aggravation of hepatitis and hepatic fibrogenesis by iron overload resulting from hemolysis.

DDS-NOH is susceptible to auto-oxidation, and the derived DDS-NO has been shown to bind covalently to protein.^77,78^ In the red blood cells, DDS-NOH is co-oxidized with hemoglobin to generate DDS-NO and methemoglobin. To reduce the toxicity of DDS, it is necessary to inhibit N-hydroxylation of DDS into rat.^79^ In this theoretical study, the binding energy results demonstrate that the dapsone molecule acts more significantly on the hepatic enzymes CYP2C9, CYP2C19, CYP3A4 and CYPE21, showing greater chemical affinity with CYPs, suggesting that its metabolism will occur independently of ALA tested. Regarding DDS-NOH, it is observed that, once present in liver enzymes, it is preferentially inhibited by the ALA in its oxidized form. DDS-NOH showed higher binding energy in all CYPs, showing a lower chemical affinity. However, no significant difference in energies is observed in the R–S configurations, which suggests that this is not relevant in the DDS-NOH inhibition.

In the process of auto-oxidation of DDS-NOH to arylnitroso derivatives, DDS-NO can be reduced to DDS-NOH by intracellular GSH. This redoxcycling leads to production of O^2•^ and other ROS, and consequently to oxidative stress.^65^ Tingle et al., to investigate the role of metabolism of DDS in the pharmacokinetics and toxicity of the compound in vivo with use of the rat, mouse and man. The authors have confirmed that DDS-NOH is N-hydroxylated by male rat liver enzymes in vitro, and that in vivo there is formation of the further metabolite, DDS-NOH glucuronide, which is excreted into bile and urine.^44^

Thus, in summary, the decrease in GSH in animals treated with DDS may be indicative of increased consumption of the thiol due to excess reactive metabolites and/or the production of ROS in the body. Indeed, other studies in animal models have also reported similar findings regarding the beneficial effects of ALA treatment. These studies have shown that ALA administration decreases serum MDA concentration, increases enzymatic antioxidant activity, and enhances nonenzymatic antioxidant levels, including GSH.^80–82^ ALA treatment has been shown to improve gene expression of glutathione reductase, which is involved in the regeneration of GSH, thus contributing to the restoration of GSH levels.^82,83^ In vitro studies with isolated human erythrocytes have also demonstrated the protective effects of ALA. It decreases susceptibility to oxidation, protects against peroxyl radical-induced hemolysis, and enhances GSH synthesis.^55,84–86^ These findings further support the beneficial role of ALA as an antioxidant and its potential to counteract oxidative damage. Overall, the data suggests that DDS treatment disrupts the antioxidant system, leading to decreased activity of SOD enzymes. However, post-treatment with ALA can reverse these effects, restoring the oxidative balance and providing direct antioxidant protection against ROS and free radicals.

The total antioxidant capacity represents the collective action of various antioxidant molecules and can be considered a dynamic balance between antioxidants and pro-oxidants. In this context, the protective effect of ALA observed in our study may be attributed to its ability to restore GSH levels and its direct and indirect scavenging of free radicals systemically and in tissues, including the liver. These findings are consistent with other studies that have reported the antioxidant and protective properties of ALA.^87,88^ Overall, our data supports the notion that ALA exerts its protective effects by restoring GSH levels, enhancing antioxidant enzyme activity, and acting as a scavenger of free radicals. In addition, ALA in its oxidized form can be inhibited DDS-NOH, how show molecular docking. These findings further highlight the potential therapeutic applications of ALA in combating oxidative stress-related conditions and protecting against cellular and tissue damage.

## 5 Conclusions

Our study conducted in an animal model has provided novel insights into the beneficial effects of post-treatment with ALA in restoring oxidative balance, liver histological changes, and mitigating hepatic damage induced by DDS. ALA administration effectively inhibited methemoglobin formation and lipid peroxidation in the blood, while also reducing iron accumulation and hepatic enzyme production triggered by DDS metabolism. These findings highlight the potential of ALA and its thiol derivatives as antioxidants in counteracting the harmful effects of DDS treatment. However, further investigations are necessary to understand the antioxidant mechanisms of ALA and its derivatives. The elucidation of the antioxidant properties and therapeutic potential of ALA may pave the way for the development of new strategies to prevent or alleviate oxidative damage associated with DDS treatment.

## Abbreviations

DDS: dapsone
DDS-NOH: dapsone hydroxylamine
ROS: reactive oxygen species
RNS: reactive nitrogen species
MDA: malondialdehyde
MB: methylene blue
ALA: alpha lipoic acid
DMSO: dimethyl sulfoxide
MetHB: methemoglobin
TEAC: trolox equivalent antioxidant capacity
2,2-ABTS: azino-bis­(3-ethylbenzothiazoline-6-sulfonic acid) diammonium salt
SOD: superoxide dismutase
GSH: glutathione
DTN: 5,5-dithiobis-2-nitrobenzoic acid
TBARS: thiobarbituric acid reactive substance
AST: aspartate aminotransferase
ALT: alanine aminotransferase
AP: alkaline phosphatase
H&E: hematoxylin and eosin
ANOVA: analysis of variance
DFT: density functional theory
ADT: AutoDock tools
CYP: cytochrome
Tf: transferrin
NTBI: nontransferrin-bound iron
